# Virtual Reality in the Treatment of Panic Disorder in the Last Decade: A Systematic Review

**DOI:** 10.2174/0117450179358928250123064503

**Published:** 2025-02-25

**Authors:** Lucio Gonçalves, Rafael Garcia, Laiana Quagliato, Jose Carlos Appolinario, Antonio Nardi

**Affiliations:** 1 Virtual Reality in Mental Health Laboratory (RVSM), Institute of Psychiatry (IPUB), Federal University of Rio de Janeiro, Rio de Janeiro, Brazil; 2 LABPR – Panic and Respiration Laboratory (LABPR), Institute of Psychiatry (IPUB), Federal University of Rio de Janeiro, Brazil; 3 Institute of Psychiatry (IPUB), Federal University of Rio de Janeiro (UFRJ), Av. Venceslau Bras, 71, Botafogo, Rio de Janeiro - RJ, Brasil - CEP 22290 140, Brazil

**Keywords:** Virtual reality, Virtual reality exposure therapy, Panic disorder, Agoraphobia in virtual reality, Real-world, PRISMA

## Abstract

**Introduction:**

Virtual Reality (VR) is an interactive, three-dimensional computing environment that enables individuals to experience a sense of presence as if they are immersed in a real-world setting. VR is currently being implemented in therapeutic interventions for individuals with certain mental disorders.

**Objective:**

To illustrate the implementation and evolution of VR in the treatment of panic disorder, agoraphobia, and panic disorder with or without agoraphobia over the past decade.

**Method:**

A systematic literature review was conducted based on articles retrieved from PubMed, Cochrane, and Web of Science, covering the period from 2013 to 2023. A total of 21 studies were selected after analyzing the titles, abstracts, and content in accordance with the Preferred Reporting Items for Systematic Reviews and Meta-Analysis (PRISMA) guidelines.

**Results:**

A total of 153 articles were initially selected and included in the study. The results demonstrated the evolution and increasing use of VR-based technologies for the treatment of mental disorders, including panic disorder and PDA.

**Discussion:**

The utilization of VR exposure therapy (VRET) for patients with panic disorder, agoraphobia, or PDA yielded measurable outcomes, including the evolution of VRET applications, an increase in the number of scientific articles and patients in recent years, as well as advancements in hardware devices, software, and other application methods, such as self-guided applications.

**Conclusions:**

The implementation of VRET is increasing in several regions worldwide, and its evolution is indisputable for the treatment of panic disorder, agarophobia, and PDA. Comparisons with traditional *in vivo* methods revealed that VRET yields satisfactory and promising outcomes. The continued evolution of VR technology is expected to expand its potential application in patients with these disorders.

## 1 INTRODUCTION

Virtual Reality (VR) is emerging as a revolutionary tool in mental health, offering novel strategies for the treatment of psychiatric disorders [1]. VR is a computer-generated, three-dimensional environment that enables users to immerse in a virtual world, where they can interact with the environment as though they were physically present in it [2, 3].This graphical imple-mentation ensures that the virtual world incorporates real-world aspects (virtual realism), and authentic sound (auditory realism), and enables the user to feel as though they are part of the environment (haptic realism) [4]. Head-mounted displays (HMDs) represent the majority of VR tools in current use, which facilitate what theorists define as direct, immediate action [2].

VR emerged as an exposure tool over 20 years ago, providing patients and therapists with more appealing strategies that, despite being undoubtedly effective, have been rejected and consequently underused [5].

VR makes it possible to replicate environments that serve as triggers for individual manifestations, depending on the specific disorder. It, therefore, enables the recreation of particular environments for individual disorders, enhances the replication of socio-psychological experiments, facilitates a shift in the thinking patterns regarding social interactions, allows for the examination of reciprocal information exchange, and enhances the potential for ecological validity and controlled real-life situations [6, 7].

VR is used as a “simulative tool” for recreating logically valid scenarios, thereby enabling the reproduction of alarming or critical situations, such as public speaking, with precise control over the delivery of stimuli tailored to the specific therapeutic requirement for each patient. Patients can, therefore, participate in simulated situations that would otherwise be extremely dangerous to experience in real life, thereby providing insights into the limitations of existing treatments [8].

Immersive VR enables the creation of interactive computer-generated environments that can replace real-world sensory perceptions with digitally generated stimuli, thereby producing the sensation of being physically present in novel, life-sized settings. VR has several benefits in mental health, including the generation of highly controlled, immersive environments, the personalization of therapeutic interventions, and the collection of real-time data, all of which contribute to enhancing therapeutic efficacy. One of the most significant benefits of VR is its ability to simulate immersive virtual environments that closely resemble the real world with high accuracy [9-11].

VR exposure therapy (VRET) can be categorized as a modern variant of exposure therapy designed to simulate environments in which patients are exposed to their specific fears. Therefore, VR provides an excellent strategy for the treatment of panic disorder and agoraphobia, allowing for the simulation of various situations in daily life. Although this prospect seems attractive, there is a scarcity of research on the therapeutic efficacy of VRET in panic disorder and agoraphobia, which limits its incorporation into clinical practice [12].

VR technologies are being increasingly implemented as a critical tool for the diagnosis and treatment of various mental disorders. They are regarded as a potentially revolutionary resource for psychological interventions and may be gradually integrated into regular clinical practice in the coming years [13].

Panic disorder with agoraphobia (PDA) is characterized by a fear of experiencing panic attacks or panic-related symptoms in situations where escape is perceived as difficult and is characterized by a sudden rush of anxiety that manifests as physiologic and cognitive symptoms, leading to a fear of specific situations [14, 15].

*In vivo* exposure therapy (iVET) is the most common therapeutic strategy for PDA, in which patients are exposed to stimuli in a gradual and controlled manner to shift their responses to the object or situation causing the fear. *In vivo,* therapies are associated with high costs and disruptive variables, which pose challenges to the implementation of the specific therapy. VRET can, therefore, provide a potential solution for addressing these limitations associated with iVET [16].

The present research primarily aimed to examine the applications of VR in the treatment of panic disorder over the last decade. To achieve this general objective, the specific objectives of the study were as follows: (a) to evaluate the evolution of VR applications in the last decade; (b) to assess the application of VR in the treatment of panic disorder and agoraphobia; and (c) to determine the therapeutic validity of VR in patients with PDA or those with panic disorder without agoraphobia.

## 2 METHODS

### 2.1 Study Design and Registration

This systematic review has been registered in the OSF Register under registration number OSF 2023-01-08 - 04:53 PM, and was conducted in accordance with the Preferred Reporting Items for Systematic Reviews and Meta-Analyses (PRISMA) guidelines. The meta-analysis was conducted using a keyword-based search strategy against the Medical Subject Headings (MeSH) database, using MeSH-indexed keywords. The adequacy of the study structure was further evaluated using the PRISMA checklist that served as a guide.

### 2.2 Research Strategy

We searched the PubMed, Cochrane, and Web of Science databases for articles in English published between 2013 and 2023, using individual keywords or keyword combinations with the Boolean connectives “AND” and “OR”. The included articles are provided in Fig. (**1**).

A total of 153 articles were initially selected by database search, after which the titles were examined, and 70 articles were removed. The abstracts of the remaining 83 articles were reviewed, which led to the removal of 54 studies. The remaining 29 articles were considered eligible for reading, and a total of 8 studies were subsequently eliminated after reviewing the contents, which left 21 articles for inclusion (Tables **1a**–**b** and **2**). The study was conducted at the RVSM Lab (Laboratório Realidade Virtual na Saúde Mental), affiliated with IPUB/UFRJ.

### 2.3 Inclusion and Exclusion Criteria

The articles selected herein were published in English between 2013 and 2023 and were obtained from the three aforementioned databases in accordance with the PRISMA guidelines, using the 5 keywords from this article. These articles addressed panic disorder and agoraphobia, specifically in relation to the use of VR technology. The excluded studies on VR applied exclusively to other disorders.

## 3 RESULTS

### 3.1 Selection of Articles Using the PRISMA Method

**Fig. (1):**
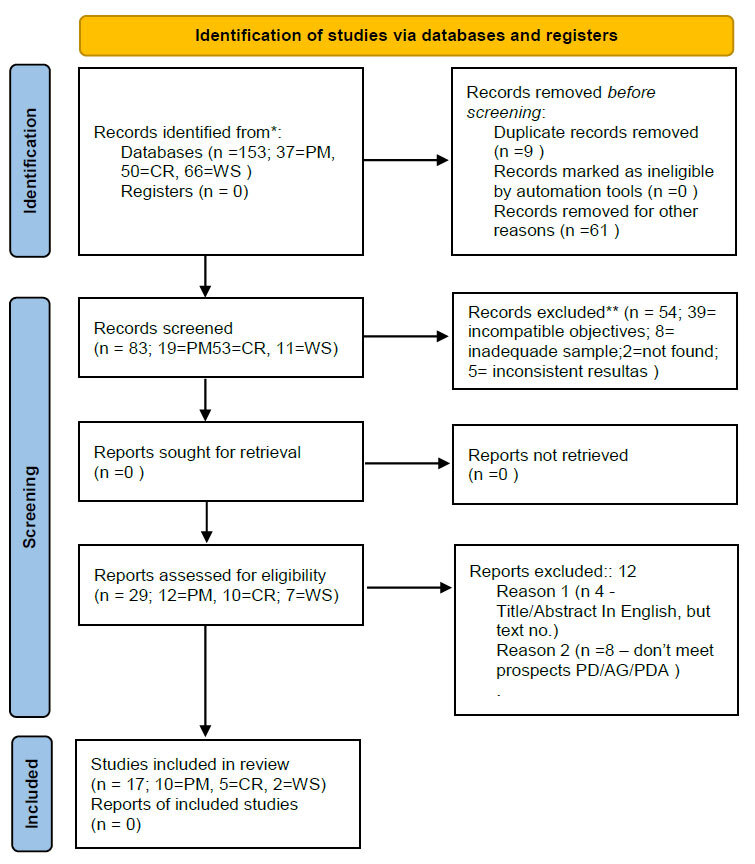
Article selection in accordance with the PRISMA guidelines.

During bibliographical research, the quantitative findings leading to the final selection of articles for qualitative synthesis are presented in accordance with the PRISMA diagram (Fig. **1**). The quantitative data extracted from the selected articles are additionally provided in Tables **1** and **2**. A total of 14 (65%), 5 (25%), and 2 (10%) articles were finally selected from the PubMed, Cochrane, and Web of Science repositories, respectively.

### 3.2 Quantitative Data

The enhanced implementation of VR techniques for mental disorders reflects the quantitative results, which are increasingly accepted by therapists and patients, which confirms the growing confidence in the application of VR-based technologies.

VR-cognitive behavioral therapy (VR-CBT) with a 360-degree video is a feasible, acceptable, and potentially effective treatment option for PDA, which is suitable in primary care settings, as indicated by a high degree of self-reported treatment satisfaction and the absence of patient dropouts [14].

It is considered that the implementation of VRET in patients with agoraphobia can significantly improve well-established psychological and psychiatric treatments [17].

Previous studies suggest that patients report satisfaction with VR-based therapy and are likely to find it more acceptable than traditional approaches. Patients with PDA find VR to be an effective strategy for treating the symptoms of this condition. Studies on VR-based exposure therapy for PDA, employing rigorous study designs and methodologies, support the efficacy of VR-based tools for the treatment of PDA [15].

The growing number of studies using VRET reinforces the quantitative results, driven by the improvement in the quality of computational resources, particularly in terms of the accuracy of images, among other factors.

It has been demonstrated that VRET is an effective therapeutic strategy for agoraphobia when implemented for 8–12 sessions, typically once a week on average, for at least fifteen minutes. Numerous studies on the implementation of VRET for phobias collectively facilitate the development of specific recommendations for the optimal implementation of VRET, thereby promoting therapeutic efficacy [18].

The computer systems used for the implementation of VRET can effectively induce panic, anxiety, hyper-ventilation, and electrodermal responses in patients with PDA but do not produce these effects in healthy control subjects [19].

It has been demonstrated that VR-based experiments can effectively replicate realistic spatial and social behaviors. VR additionally allows for experimental control and systematic variations that would be difficult in real-world scenarios [20]. Studies have additionally revealed that VRET yields positive outcomes in the treatment of most phobias; however, the results may vary depending on the specific disorder and resources employed [21].

PM, PubMed; CR, Cochrane; WS, Web of Science.

Quantitative distribution of research articles selected from the three academic databases, and the steps employed for verification prior to final inclusion (Tables **1** and **2**).

The numbers corresponding to each database (PM, CR, and WS) represent the sum of articles obtained through independent keyword-based searches, using each of the five keywords individually.

A total of 3 included articles (14%) were published in the first half of the last decade (2013–2017), while 18 (86%) articles were published in the second half (2018–2023).

### 3.3 Brief Description of Selected Articles

The 21 articles finally selected based on their content, specifically relating to PD and agoraphobia, are briefly described hereafter.

### 3.4 Demographic Analysis

Only 3 countries were represented by the selected articles published between 2013 and 2017, while 12 countries were represented between 2018 and 2023, of which 11 did not appear in the studies published between 2013 and 2017.

The number of articles on the application of VR in mental health was analyzed by dividing the decade into two halves (2013–2017 and 2018–2023), which revealed an exponential growth in the number of articles published over the ten years studied herein. It was observed that of the 21 articles selected in this study (including 14, 5, and 2 articles from PubMed, Cochrane, and Web of Science, respectively), the number of articles on the use of VR in mental health therapy increased by more than 500%, from 3 to 18 articles, from the first half of the decade to the second half, reflecting a growing interest in this field within the scientific community.

The literature review by Yeung (2021), covering 28 years from 1992 to 2019, mapped the number of annual publications on VR in the field of medicine, which revealed a substantial increase in the number of published articles on VR as well as the number of citations. Analysis of the number of publications from 2013 to 2019, which falls within our temporal range of 2013–2023, revealed a four-fold increase from 300 to 1250 publications per year, while the number of annual citations on VR increased from 450 to 2000 citations per year [2].

### 3.5 Therapeutic Efficacy

The application of VR offers several therapeutic advantages for patients with mental disorders, especially in terms of outcomes, compared to *in vivo* therapies. Despite the absence of consistent confirmation, the outcomes described in the selected articles on the use of VR with computers, 3D glasses with supervision, and self-guided methods are generally positive.

The efficacy of CBT in the absence of a virtual therapist remains to be investigated; however, a virtual therapist appears to be an essential component of fully self-guided VR therapy [22]. On the other hand, the use of VRET in CBT-based psychological therapies for panic disorder is less efficacious in certain cases, which is possibly attributed to the technological resources employed [23].

## 4 DISCUSSION

VR is emerging as a revolutionary tool in mental health, providing a novel strategy for the treatment of psychiatric disorders [1].

There is substantial consensus regarding the advantages of applying VR in mental health disorders, particularly for the treatment of individuals with panic disorder and agoraphobia. These advantages include enhanced therapeutic control compared to *in vivo* or self-guided processes, as well as lower treatment costs. It has been additionally reported that VRET yields positive therapeutic outcomes in patients with agoraphobia [24].

However, a significant number of patients remain untreated due to limited therapeutic resources, and digital self-guided short-term treatment applications may help address this issue; however, there is a scarcity of data on their efficacy [22, 25]. It is, therefore, necessary to further evaluate self-guided treatment strategies. The findings suggest that VRET does not achieve the same level of immersion and presence as iVET for certain phobias [21].

VR provides a dynamic, engaging, and interactive environment for patients suffering from certain disorders, and its benefits in mental health include the enhancement of treatment efficacy [10].

### 4.1 Geographic Expansion

The development of VR applications for the treatment of panic disorder and agoraphobia evolved from 2013 to 2022. This duration was divided into two periods in this study, namely, 2013–2017 and 2018–2023, and the findings revealed significant growth in the field across multiple countries. The articles selected from the first half of the last decade (2013–2017) represented 3 countries, while those selected from the second half represented 12 countries, of which 11 were new countries, indicating significant geographic expansion.

### 4.2 Analysis of the Number of Articles

A total of 3 and 14 articles were finally selected according to the PRISMA guidelines for the periods 2013–2017 and 2018–2023, respectively, which consti-tuted 18% and 82%, respectively, indicating a substantial growth in the number of publications.

Of the 17 studies selected herein, approximately 70% employed or analyzed studies that implemented HMDs or more accurate devices, while 30% utilized computers and represented older articles. This highlights the evolution of VR technology and demonstrates the enhancement of accuracy and presence in more recent years.

### 4.3 Participant Profiles/Target Demographics

Despite the growth in the number of articles and authors from countries that have started publishing studies on VRET, several of these articles feature small sample sizes, which reduces the ability to draw generalized conclusions regarding the outcomes of implementing VR-based thera-peutics.

Further studies with larger sample sizes are therefore necessary, as several studies have employed smaller sample sizes, which likely contributes to the inconclusive results observed in several studies [21].

### 4.4 Hardware Devices and Software

The development of software and the selection of appropriate hardware for the implementation of VRET are key discussions that require a high degree of fidelity to replicate real-world environments. The use of VRET should reinforce the concepts of presence and customization for enhancing its efficacy and for stabilizing the evidence base for VR-based clinical therapies [26]. VR technologies can potentially enrich and reinforce therapies, providing a more engaging, personalized, and effective diagnostic and therapeutic experience for patients [27].

### 4.5 Therapeutic Validity

Therapeutic validity is typically assessed based on various factors, including the classification of the disorder to be treated, its stage, the strategy used for evaluating treatment outcomes, and various demographic factors such as age group, patient profile, and technologies employed, among others.

VR offers unlimited opportunities that enable the adaptation of specific approaches to complex problems and facilitate the individualization of interventions. Meta-analyses of articles on the implementation of VR for the treatment of panic disorder revealed highly significant outcomes [24, 28].

Another meta-analysis demonstrated that the treat-ment outcomes of VR are comparable to those of conven-tional treatments and are transferable and generalizable to real-life situations [20].

VRET has been extensively investigated and applied for the treatment of anxiety disorders. Previous studies have demonstrated the efficacy of VR in generating exposure scenarios that simulate real-world situations, leading to a reduction in anxiety symptoms. It has been reported that VRET is more effective in reducing disease symptoms compared to the individuals in the waitlist group [29].

VR displays immense therapeutic potential and researchers are of the opinion that highly standardized exposure procedures should be utilized for achieving therapy [12].

It has been confirmed that VR-based therapy for panic disorder displays significant correlations with assessments conducted without VR. The application of VR for the treatment of panic disorder has demonstrated its validity as a potential therapeutic strategy for this disorder [30].

VR has been explored as a promising strategy for addressing body image distortion and dysfunctional eating behaviors in individuals with eating disorders [31].

Another study demonstrated that VRET yields positive outcomes during the treatment of most phobias, although results can vary depending on the specific disorder and the resources employed [21]. Among the various factors, the age group of patients can significantly influence the evolution and efficacy of VR-based therapy. The application of VR-based treatment in children and adolescents within therapeutic contexts remains an aspect that has been scarcely investigated to date. VRET is a potentially effective approach for exposure-based therapy, especially for adolescents, due to its low barriers and incorporation of engaging elements; however, there is a scarcity of research in the area [12]. The treatment of adolescent patients, combined with the rapid evolution of VR technology, can accelerate the implementation of this technology for the therapeutic benefit of panic disorder and other mental conditions.

Therapeutic validity also encompasses the utilization of self-guided digital VR in the absence of face-to-face therapists interacting virtually, and sometimes even without any online therapist. The expansion of VRET is still in its initial and exploratory stages, and further studies are necessary to confirm its therapeutic validity. The implementation of self-guided VR yields inconsistent results and has been shown to have limited therapeutic efficacy [25].

### 4.6 Limitations

Temporal delimitations can impose limitations when researching VR-based technologies, as the articles from the beginning of the decade, although valuable, may have certain limitations that could already have been resolved by the end of the decade. In several cases, the articles selected from 2013 to 2017 are based on technical alternatives that evolved after this period. This limitation was mitigated by the significantly higher number of selected articles (82%) in the second half of the decade. The use of CS on a regular TV screen, combined with the lack of interactivity, can pose a limitation [21]. However, these limitations were resolved in the second half of the decade by implementing VRET using 3D glasses.

Another limitation is the use of protective factors, such as counseling interventions, which are frequently implemented without prescriptions for preventing or alleviating disease symptoms. However, this aspect was not explored as it was beyond the scope of this study.

The application of VR for the treatment of panic disorder has been less thoroughly researched compared to other conditions, such as anxiety disorder. However, there has been a notable increase in the number of studies on the application of VRET for the treatment of panic disorder in recent years, confirming positive developments in this field.

VRET is most frequently implemented in universities, irrespective of the type of disorder, which is advantageous for the further development of VRET in mental health. However, the evolution of VRET remains limited in clinical practice, which restricts an exploration of its outcomes. Although this scenario does not pose a limitation to the advancement of the study, it is nevertheless structured to facilitate its progression in the medical field.

The limitations of the selected articles were resolved over the years owing to the evolution of VR-based technologies from the beginning of the decade to the present.

## CONCLUSION

Digital technologies have become an integral part of our daily lives. The landscape of modern VR has evolved significantly compared to the VR systems used in the majority of studies published prior to 2018. Significant progress has been recorded since 2018 in terms of the increase in the number of studies, number of countries involved, sample sizes, and patient outcomes. It has been reported that VRET has received positive responses from participants compared to traditional iVET, making it an attractive alternative therapy. However, it should be noted that cost and performance issues may depend on the specific disorder targeted for therapy. It has been suggested that patients are satisfied with VR-based therapy and may find it more acceptable than traditional approaches.

Previous studies provide evidence supporting the positive outcomes of VR-based therapy in psychiatric disorders; however, its efficacy depends on the specific area of the study.

Numerous studies on the use of VR-based exposure therapy for phobias, in general, have allowed for the formulation of recommendations for the implementation of VRET, thereby enabling therapeutic efficacy. It has been demonstrated that VR-based therapy is an effective therapeutic strategy for panic disorder, and its outcomes are superior to those of standard interventions.

Despite the technological complexity, it is expected that VR-based therapy will experience significant growth within the field of mental health, as indicated by its evolution over the last decade, an increase in the number of published articles, and the growing number of countries engaged in research on VR-based therapy.

It has been demonstrated that the efficacy of VR-based strategies for the treatment of panic disorder and the outcomes are superior to those of standard interventions.

The future of research on mental disorders appears promising, with the implementation of VR poised to transform diagnostic and treatment practices while potentially influencing mental health policies.

## Figures and Tables

**Table 1 (a):** Quantitative distribution of research articles.

**Databases**	**Number of articles initially obtained**	**Number of articles eliminated based on the title**	**Number of articles selected for abstract** **review**	**Number of articles eliminated based on abstract review**	**Number of articles eligible for reading**	**Number of articles eliminated after reading**	**Number of articles finally included for analysis**
**PubMed**	37	18	19	03	16	02	**14**
**Cochrane**	66	13	53	43	10	05	**05**
**Web of Science**	50	39	11	04	07	05	**02**
**Total**	153	70	83	50	33	12	**21**

**Table 1 (b):** Quantitative distribution of included research articles.

**Databases**	Included Articles	**Year of Publication**										
2023	2022	2021	2020	2019	2018	2017	2016	2015	2014	2013
**PubMed**	**14**	02	03	04	02	0	01	01	0	01	0	0
**Cochrane** **Web of** **Science** **Total**	**05** **02** **21**	0 01 03	02 0 05	01 01 06	0 0 02	0 0 0	01 0 02	0 0 01	0 0 0	0 0 01	0 0 0	01 0 01

**Table 2:** Quantitative distribution of included articles published between 2013 and 2023.

Authors/Article/Location/Database/Refs	**Objectives**	**Methodology**	**Sample/SDF**	Synthesis
Freitas JRS et al., 2021, PubMed, Portugal [21]	To compare the relative efficacy of VRET versus iVET in individuals suffering from phobias	Literature review	S = 30 articles in the final selection	Results demonstrated that VRET has positive outcomes in the treatment of PDA, but further research with larger sample sizes is necessary
Weibel RP, 2018, PubMed, Switzerland [20]	To describe a protocol that employs the experiments in virtual environment (EVE) framework to conduct navigation experiments in VR using physiological sensors	Ethics Commission of ETH Zurich	Not available	VR experiments allow for a more precise measurement of behavioral and psychological data. VR allows experimental control and systematic variations that would be difficult in real-life situations
Maples-Keller JL et al., 2017, PubMed, USA [15]	To review existing literature on the therapeutic efficacy of VR, with an emphasis on PDA and anxiety disorders	Literature review	S = 28 individuals	Patients with PDA report satisfaction with VR-based therapy and may find it more acceptable than traditional approaches
Pitti CT, 2015, PubMed, Spain [17]	To evaluate how patients with agoraphobia cope with phobic stimuli	A total of 99 individuals were divided into 3 groups, of which one was treated with VR	S = 99 individuals diagnosed with agoraphobia	The implementation of VRET for the treatment of patients with agoraphobia can enhance psychological and psychiatric interventions
Krzystanek, 2021, PMID = 34621197, Poland [18]	To assess the effective implementation of exposure therapy in a VR environment	Systematic review	S1 = 362 reports, S2 = 49 selected reports, 11 reports on agoraphobia	VRET is effective for the treatment of agoraphobia when implemented for 8–12 sessions. A large number of studies on VRET allows for the formulation of recommendations on its implementation for enhancing therapeutic efficacy
Lunden J, 2022, PubMed, Sweden [14]	To investigate whether VR-CBT can be implemented for the treatment of panic disorder in primary care settings	Cross-sectional research	S = 12 in 10–12 weeks	VR-CBT appears to be a feasible, acceptable, and potentially effective therapeutic strategy for PDA. The patients reported high satisfaction with the procedure and a low rate of dropout
Freire et al., 2020, PubMed, Brazil [19]	To ascertain whether a given computer simulation (CS) is capable of inducing panic disorder, anxiety, and psychophysiological changes in patients with PDA	Longitudinal research	S = 30 patients with PDA and N = 30 healthy patients	Exposure to the CS produced effects similar to *in vivo* exposure, including respiratory and caffeine challenges. Further studies are necessary for comparing the various forms of PDA treatment
Wiebe et al., 2022, PubMed, Germany [13]	To review existing literature on the application of VR in the diagnosis and treatment of mental disorders	Systematic review	S1 = 9315 studies and S2 = 721 reports	The increasing implementation of VR technologies is both real and significant and exhibits immense potential for the diagnosis and treatment of mental disorders
Yeung, 2021, PubMed, China, Austria, USA, Sweeterland Netherlands, Poland, and Bulgaria [2]	To identify and analyze the growth of scientific literature on the use of VR in medicine	Review of literature published between 1992 and 2019 on the growth of literature on VR	S = 8399 articles	The number of publications on VR in the field of medicine has increased exponentially, with projections in the last decade being 3–4-fold higher than those in the previous two decades
Dellazizzo et al., 2020, PubMed, Canada [28]	To summarize the available literature on the efficacy of VR-based interventions for various psychiatric disorders	Meta-revisions from PubMed, PsycInfo, Web of Science, and Google Scholar. The included articles qualitatively examined the therapeutic efficacy of VR-based interventions for psychiatric disorders	S = 11 articles selected through meta-analysis from the 322 reports initially selected	Evidence suggests that VR-based interventions yield medium to large overall effects when compared to inactive controls
Meyerbrook and Morina, 2021, Cochrane, Netherland and Germany [12]	To discuss various clinical aspects, including the integration of VR in clinical practice and potential future applications	Narrative review	Not available	VRET is a potentially effective approach for exposure therapy, particularly in adolescents, due to its low barriers and the incorporation of engaging elements. Given their connection with a technology-driven environment, the application of VRET in adolescents is assumed to potentially enhance treatment, early intervention, and disease prevention
Roh and Jung, 2022, Cochrane, South Korea [26]	To investigate existing literature for evaluating the efficacy of the presence and keyword customization in VR-based exposure therapy for panic disorder and agoraphobia	All the participants were exposed to the same VR scenario, and the environment was randomized for each participant. The participants were interviewed to identify more vulnerable contexts or situations that provoke panic	S = 25 studies	For patients with PDA, the groups that used HMD and customized VR technologies showed superior outcomes compared to the group that did not use these resources
Planert et al., 2022, Cochrane, Germany [25]	To investigate the efficacy of self-guided treatment combining psychoeducation and VRET	The diagnostic process was divided into 2 sections: 6 weeks of psychoeducation plus VRET, and an additional 4 weeks of self-guided VRET	S = 30 patients with panic disorder, agoraphobia, and PDA	Despite limitations in the efficacy of self-guided treatment, digital self-guided short-term applications may help address the issues of patients not having access to therapy
Malbos et al., 2013, Cochrane, Australia [24]	To evaluate the independent effects of VRET on agoraphobia	Controlled study. The participants were assigned to 2 groups: VRET only group and VRET with cognitive therapy group	S = 18 patients with agoraphobia	VRET displayed proven positive outcomes in patients with agoraphobia
Pampoli, 2018, Cochrane [23]	To examine whether specific components and their combinations are superior to other therapeutic strategies for panic disorder	Meta-analysis of articles from Medline, EMBASE, PsychInfo, and Cochrane	S1 = 2526 articles and S2 = 72 selected articles, with 4064 participants	Effective CBT packages for panic disorder should include both face-to-face and interoceptive exposure components, excluding muscle relaxation and VR, which displayed the least efficacy
Shin et al., 2021, Web of Science, Republic of Korea [22]	To provide data regarding the efficacy of mobile app-based, self-led VR-CBT for the treatment of panic disorder	Treatment with VR and a waitlist group for 4 weeks across 12 sessions	S = 54 patients with panic disorder	Self-guided mobile app-based VR reduced the symptoms of panic disorder and restored the autonomic nervous system. However, the implementation of self-guided therapy requires extensive research, and its safety and efficacy warrant further discussion
Byeing-Hoon, 2023, Web of Science, Republic of Korea [30]	To evaluate the feasibility of VR-based techniques for assessing panic disorder	The study used four models based on the main components of TCC, followed by comparative analyses of the 2 groups	S = 25 patients with panic disorder and 28 healthy individuals	The study provided preliminary evidence that the VRA-PD can be implemented as a valid tool for assessing anxiety (panic disorder) behaviors
Oh S et al., 2024, Front Psychiatry, Republic of Korea [27]	To use VR and artificial intelligence for the diagnosis of ADHD	Exploratory study	S = 21 children aged 6–12 years, of which 5 children had ADHD	VR can potentially enrich and refine therapies, offering a more engaging, personalized, and effective diagnostic and therapeutic experience
Tan YL et al., 2024, Anxiety Stress Coping, China [29]	To describe the state of the art of VRET for social anxiety disorder with meta-analysis and meta-regression	Meta-analysis and meta-regression based on PRISMA guidelines	S = 10,643 manuscripts, of which 20 were finally selected	The application of VRET in anxiety disorders demonstrated that VR can effectively replicate exposure scenarios simulating real-world situations, leading to a reduction in anxiety symptoms
Riva G et al., 2021, Clin. Psycholol Psychother., Italy [8]	To demonstrate the use of VR in the treatment of eating disorders	Narrative review	Not applicable	VR can offer a promising strategy for addressing body image distortion and dysfunctional eating disorders
Slater M et al., 2022, Front. Virtual Real., USA [10]	To review the concept of presence in VR, which is typically conceived as a sense of “being there” in the virtual world	Literature review	Not applicable	Presence consists of two orthogonal illusions referred to as place illusion (PI, which represents the illusion of being in the place and is depicted by the VR) and plausibility (Psi, which denotes the illusion that the virtual situations and events are actually occurring)

Elements of the 21 articles that were finally selected based on PRISMA guidelines

AG, agoraphobia; CBT, cognitive behavioral therapy; CS, computer simulation; EVE, experiments in virtual environment; iVET= *in vivo* exposure therapy; HMD, head-mounted display; MH, mental health; PA, panic attack; PD, panic disorder; PDA, panic disorder with agoraphobia; RCT, randomized controlled trial; S, sample; VR, virtual reality; VRA-PD; VRET, VR exposure therapy.

## Data Availability

All the data and supporting information are provided within the article.

## LIST OF ABBREVIATIONS

VR: Virtual Reality
HMDs: Head-mounted Displays
PDA: Panic Disorder with Agoraphobia
iVET: *In vivo* Exposure Therapy
VRET: VR Exposure Therapy
VR-CBT: VR-cognitive Behavioral Therapy

## SUPPLEMENTARY MATERIALS

The supplementary materials are available on the publisher’s website along with the published article.
